# Supplementary material for: Mobilization of multilineage-differentiating stress-enduring cells into the peripheral blood in liver surgery
Source: PLoS One. 2022 Jul 21;17(7):e0271698. doi: 10.1371/journal.pone.0271698 (PMC9302816; doi:10.1371/journal.pone.0271698)
Supplement: S1 Appendix — (DOCX) [file pone.0271698.s001.docx]

**Materials and methods**

**Plasma sphingosine-1-phosphate (S1P) concentration**

Blood samples were obtained from 15 patients (MHR; n = 10, mhr; n = 5). Blood was centrifuged (10,000 *g* for 15 min), and plasma was collected and frozen at −80 °C until plasma S1P concentration was analyzed. The analyses were conducted using liquid chromatography tandem mass spectroscopy (API 4000™ AB/MDS; Sciex, Framingham, MA, USA) at the Toray Research Center (Kamakura, Japan).

**Results**

**Plasma S1P concentration and Muse cell number**

In 15 patients whose plasma S1P concentration was analyzed, the PB-Muse cell number increased after surgery (before surgery 285.5 ± 306.9 cells/100µL, POD3 1258.8 ± 1652.2 cells/100µL, POD7 674.5 ± 757.3 cells/100µL, S1 Fig. A); however, plasma S1P concentration showed different dynamics from PB-Muse cells (before surgery 338.3 ± 59.4 ng/mL, POD1 291.5 ± 78.5 ng/mL, POD3 319.4 ± 57.4 ng/mL, POD7 318.3 ± 51.5 ng/mL, S1 Fig. B). Plasma S1P levels (maximum S1P levels on PODs 1, 3, and 7) did not differ between the two groups (MHR 361.5 ± 67.1 ng/mL, mhr 326.4 ± 41.2 ng/mL, S1 Fig. C). The maximum PB-Muse cell number did not correlate with plasma S1P levels (*p* = 0.640; S1 Fig. D).
