# Supplementary figures and images for: Mobilization of multilineage-differentiating stress-enduring cells into the peripheral blood in liver surgery
Source: PLoS One. 2022 Jul 21;17(7):e0271698. doi: 10.1371/journal.pone.0271698 (PMC9302816; doi:10.1371/journal.pone.0271698)

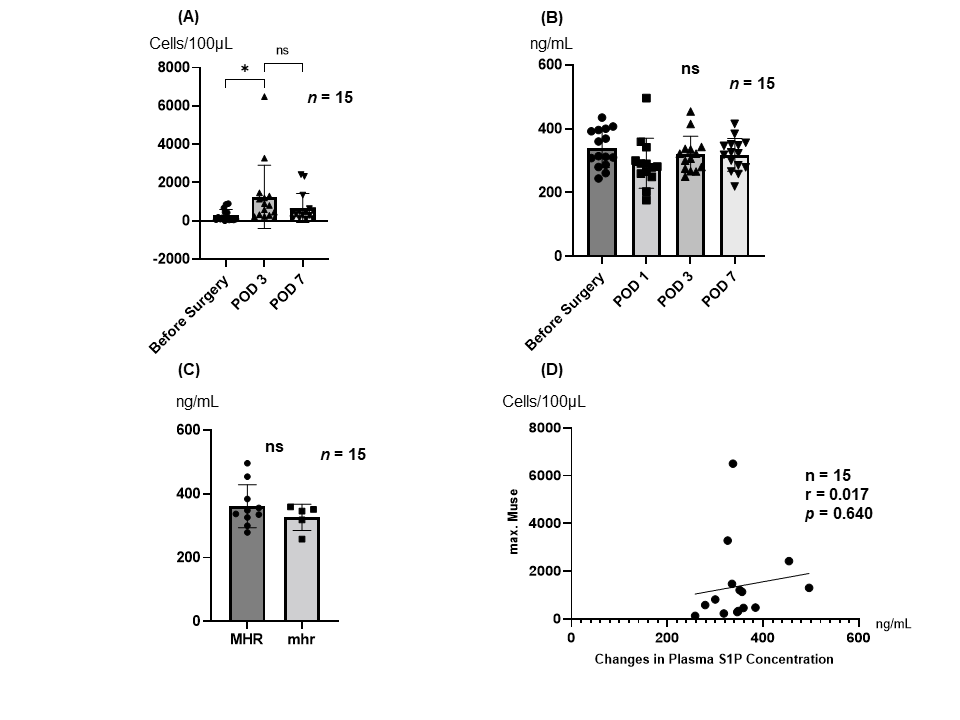

Supplement: S1 Fig — A: Changes in the number of PB-Muse cells after hepatectomy. B: Changes in plasma S1P concentrations after surgery. C: Plasma S1P levels (maximum S1P levels on PODs 1, 3, and 7) in the MHR and mhr groups. D: Relationship between plasma S1P levels and the Max PB-Muse. S1P = sphingosine-1-phosphate. *p <0.05. (TIF) [file pone.0271698.s002.tif]
