# Supplementary material for: Mobilization of multilineage-differentiating stress-enduring cells into the peripheral blood in liver surgery
Source: PLoS One. 2022 Jul 21;17(7):e0271698. doi: 10.1371/journal.pone.0271698 (PMC9302816; doi:10.1371/journal.pone.0271698)
Supplement: S1 Table — (DOCX) [file pone.0271698.s003.docx]

**S1 Table. Comparison of the preoperative PB-Muse cell numbers affecting Smoking**

|  | **Smoker (n = 9)** | **nonsmoker (n = 38)** | ***p*-value** |
| --- | --- | --- | --- |
| preoperative PB-Muse (cells/100 μL) | 529.4 ± 602.3 | 362.8 ± 421.6 | 0.209 |
