# Supplementary material for: Mobilization of multilineage-differentiating stress-enduring cells into the peripheral blood in liver surgery
Source: PLoS One. 2022 Jul 21;17(7):e0271698. doi: 10.1371/journal.pone.0271698 (PMC9302816; doi:10.1371/journal.pone.0271698)
Supplement: S2 Table — (DOCX) [file pone.0271698.s004.docx]

**S2 Table.** **Comparison of the preoperative PB-Muse cell numbers affecting Drinking**

|  | **Drinker (n = 17)** | **nondrinker (n = 30)** | ***p*-value** |
| --- | --- | --- | --- |
| preoperative PB-Muse (cells/100 μL) | 451.4 ± 589.8 | 362.7 ± 372.8 | 0.808 |
