# Supplementary material for: Mobilization of multilineage-differentiating stress-enduring cells into the peripheral blood in liver surgery
Source: PLoS One. 2022 Jul 21;17(7):e0271698. doi: 10.1371/journal.pone.0271698 (PMC9302816; doi:10.1371/journal.pone.0271698)
Supplement: S3 Table — (DOCX) [file pone.0271698.s005.docx]

**S3 Table. Effect size of analysis with significant difference**

|  | ***p*-value** | ***Effect size*** |
| --- | --- | --- |
| Table 1. HCC of diagnosis | 0.008 | 0.39 |
| Table 2. Surgical approach | 0.038 | 0.30 |
| Table 2. Operation time | 0.001 | 0.59 |
| Table 2. Blood loss | 0.002 | 0.44 |
| Table 2. Max. WBC | 0.044 | 0.29 |
| Table 2. Max. T-Bil | 0.005 | 0.41 |
| Table 2. Length of hospital stay | 0.004 | 0.42 |
| Table 3. Surgical approach | < 0.001 | 0.52 |
| Table 3. ΔMuse | 0.043 | 0.29 |
| Fig. 3A | 0.008 | 0.39 |
| Fig. 3B Before Surgery vs. POD3 (no complication cases) | 0.002 | 0.45 |
| Fig. 3B POD3 vs. POD7 (no complication cases) | 0.044 | 0.29 |
| Fig. 4C | 0.033 | 0.31 |
| Fig. 5B | 0.043 | 0.29 |
| Fig. S1A | 0.002 | 0.82 |

HCC, hepatocellular carcinoma; max. WBC, maximum white blood cells on PODs 3 and 7; max. T-Bil, maximum total bilirubin on PODs 3 and 7
