# Supplementary material for: Mobilization of multilineage-differentiating stress-enduring cells into the peripheral blood in liver surgery
Source: PLoS One. 2022 Jul 21;17(7):e0271698. doi: 10.1371/journal.pone.0271698 (PMC9302816; doi:10.1371/journal.pone.0271698)
Supplement: Attachment — Submitted filename: PONE revision.docx [file pone.0271698.s006.docx]

Muse cells are pluripotent non-tumorigenic cells with high capacity of tissue regeneration and function recovery. In addition, Muse cells are endogenous cells present in connective tissues (i.e. bone marrow, adipose tissue) that go into circulation (PB-Muse cells) to repair damage tissue when an injury occurs as it was demonstrated in patients undergoing cerebral stroke and acute myocardial infarction.

In this manuscript Kikuchi et al., analyzed Muse cells mobilization into the peripheral blood in patients with liver tumors that underwent major or minor hepatic resection (MHR and mhr, respectively) at days 3 and 7 post-surgery. The number of circulating Muse cells significantly increased in the MHR vs mhr patients. In addition, there was a positive correlation between circulating Muse cells and recovery of liver volume after surgery.

This is an interesting paper, with novel findings which could have implications in the process of liver regeneration after hepatectomy through the mobilization and repair capacity of endogenous Muse cells.

There are some issues that need to be clarified:

1- Fig. 3D and 3E show values of a patient with max Muse cell>10,000 and delta Muse >120 that look to be outliers. How was the liver recovery and AST values of this particular patient before and at days 3 and 7 after surgery?
2- Remove Fig 5 because of lack of power (only n=15). Include these results in Discussion as data not shown.
3- If S1P is not a major player in mobilization of Muse cells, what other mechanism(s) could be proposed?

Minor changes:

1- The terms maximum PB-Muse cell number and maximum AST are quite confusing, both need to be replaced.
2- Include in the reference section paper by Heneidi et al, 2013.
