# Supplementary material for: Mobilization of multilineage-differentiating stress-enduring cells into the peripheral blood in liver surgery
Source: PLoS One. 2022 Jul 21;17(7):e0271698. doi: 10.1371/journal.pone.0271698 (PMC9302816; doi:10.1371/journal.pone.0271698)
Supplement: Attachment — Submitted filename: Reply letter.docx [file pone.0271698.s008.docx]

Dr. Emily Chenette

Editor-in-Chief

Dr. Chengming Fan

Academic Editor

***PLOS ONE***

June 29, 2022

PONE-D-22-04405

Mobilization of multilineage-differentiating stress-enduring cells into the peripheral blood in liver surgery.

Thank you very much for the opportunity to revise our manuscript. The reviewer's comments were very helpful. We have corrected the manuscript point-by-point and have provided responses to each comment below. We believe that the manuscript has been substantially improved, and we would be grateful if you would consider its suitability for publication in *PLOS ONE*. We look forward to hearing from you.

Sincerely,

Koji Kikuchi M.D.

Department of Surgery,

Iwate Medical University School of Medicine,

2-1-1 Idai-dori, Yahaba, Iwate, 028-3609, Japan

Response to Reviewer #1:

***1. Since bar graphs were changed to plots, now the data became interpretable. On the other hand, values for average and SD overlapped with plots in the revised figures. For this, the referee asks authors to describe numerical values of average and SD in the main text, so that readers may understand the meaning of data. For example, page 11, second and third paragraphs, sentences for Fig 2 and Fig 3 needs numerical values for each group, neoplasm and healthy groups.***

Reply:

In accordance with your suggestion, we have added numerical values for the average and SD in the Results section (Page 11, Lines 12), (Page 11, Lines 13), (Page 11, Lines 15), (Page 11, Lines 17), (Page 12, Lines 3), (Page 12, Lines 5), (Page 12, Lines 8), (Page 13, Lines 5), (Page 13, Lines 6), (Page 13, Lines 7), (Page 13, Lines 8), (Page 13, Lines 14) and (Page 13, Lines 17).

***2. looking at Fig 2A and 2B, one single plot in neoplasm group seem to elevate the average in neoplasm group. They stated in the text that “the number of PB-Muse cell was higher in the group with both benign and malignant neoplasms (n =55) than in the healthy group, which included four liver donors (n = 14, p = 0.056; Fig. 2A).” Since there was no statistical difference, authors should not carelessly state higher. “High but without statistical significance” might be better sentence.***

***The same issue falls into other “higher” parts throughout the text.***

***Furthermore, authors should check the*** ***appropriateness of the single dot in the neoplastic/metastatic patients in Fig 2 to be included as a proper sample. This single dot seems to distorts the data that it should be.***

Reply:

Thank you very much for your invaluable comments. We have changed the indicated sentences (Page 2, Lines 9), (Page 11, Lines 11), (Page 12, Lines 7), (Page 13, Lines 6), (Page 15, Lines 13) and (Page 16, Lines 7). In the case of the single dot in Fig. 2, that point has been excluded as an outlier because the number of PB-Muse cells before surgery was greater than +/- three times the SDs. We have added explanatory text regarding outliers in the Materials and methods section (Page 8, Lines 1) and Results section (Page 11, Lines 10).

***3. page 11, third paragraph: why authors can*** ***conclude that POD3 was peak in Fig 3A. There was no statistical significance between POD3 and POD7 (p=ns). The same problem falls onto Fig 3B, as well. How POD7 can be the highest while POD3 and POD7 were ns.***

***Again a single plot in Fig 3C seems to be extraordinal case and thus is questionable to be included as one of the rational samples.***

Reply:

As suggested by the reviewer, we cannot conclude that POD3 was a peak in Fig 3A and POD7 was the highest in Fig 3B. We have changed the indicated sentence (Page 11, Lines 17) and (Page 12, Lines 2). In the case of the single dot in Fig. 3C, that point has been excluded as an outlier because Max PB-Muse was greater than +/- three times the SDs. We have added explanatory text regarding outliers in the Materials and methods section (Page 8, Lines 1) and Results section (Page 8, Lines 13). We have also changed the data throughout the text after reanalysis.

***4. page 13, top line: the MHR group (1,852.0 ± 2,431.8 cells/100μL) was higher than in the mhr group (847.4 ± 1,047.9 cells/100μL, p = 0.051; Fig. 4B -> higher but without statistical significance.***

Reply:

In accordance with your suggestion, we have changed the indicated sentence (Page 13, Lines 6).

***5. page 11: “ΔMuse was significantly higher in patients with ΔVolume ≥ 1.19 (16.3 ± 25.2) than in those with ΔVolume ˂1.19 (8.4 ± 26.4, p = 0.043; Fig. 5B). ΔMuse did not correlate with ΔVolume (p = 0.917; Fig. 5C).” -> The two sentences are inconsistent. Needs to be explained or reconsider the statement.***

Reply:

Thank you for providing these insights. Although Fig. 5C showed a correlation, and not a relation between cause and effect, as you pointed out, the two sentences are too confusing. We have therefore removed Fig. 5C and the relevant sentence from the Results section and Discussion sections.

***6. Table 2 is sloppy. Extra lines should be deleted.***

Reply:

Thank you for your comment. As you pointed out, this is a mistake. The extra lines have been deleted.

Response to Reviewer #2:

***1.*** ***Figures 3D/E have to be redone without including the outlier. P values needs to be re-calculated and results and discussion needs to modified accordingly.***

Reply:

Thank you very much for your important comments. A case of Max PB-Muse greater than +/- three times SDs has been excluded as an outlier. We have performed a reanalysis that excluded this case. After this reanalysis, the Max PB-Muse and the ΔMuse did not correlate with the Max AST. Therefore, we have removed Fig. 3D and E and the relevant sentences from the Results section and Discussion sections.
